# PLM-ATG: Identification of Autophagy Proteins by Integrating Protein Language Model Embeddings with PSSM-Based Features

**DOI:** 10.3390/molecules30081704

**Published:** 2025-04-10

**Authors:** Yangying Wang, Chunhua Wang

**Affiliations:** College of Information Technology, Shanghai Ocean University, Shanghai 201306, China; 2252104@st.shou.edu.cn

**Keywords:** autophagy proteins, position-specific scoring matrix, protein language model, support vector machine

## Abstract

Autophagy critically regulates cellular development while maintaining pathophysiological homeostasis. Since the autophagic process is tightly regulated by the coordination of autophagy-related proteins (ATGs), precise identification of these proteins is essential. Although current computational approaches have addressed experimental recognition’s costly and time-consuming challenges, they still have room for improvement since handcrafted features inadequately capture the intricate patterns and relationships hidden in sequences. In this study, we propose PLM-ATG, a novel computational model that integrates support vector machines with the fusion of protein language model (PLM) embeddings and position-specific scoring matrix (PSSM)-based features for the ATG identification. First, we extracted sequence-based features and PSSM-based features as the inputs of six classifiers to establish baseline models. Among these, the combination of the SVM classifier and the AADP-PSSM feature set achieved the best prediction accuracy. Second, two popular PLM embeddings, i.e., ESM-2 and ProtT5, were fused with the AADP-PSSM features to further improve the prediction of ATGs. Third, we selected the optimal feature subset from the combination of the ESM-2 embeddings and AADP-PSSM features to train the final SVM model. The proposed PLM-ATG achieved an accuracy of 99.5% and an MCC of 0.990, which are nearly 5% and 0.1 higher than those of the state-of-the-art model EnsembleDL-ATG, respectively.

## 1. Introduction

Autophagy is an evolutionarily conserved and highly regulated lysosomal pathway that facilitates the degradation of macromolecules such as proteins, glycogen, lipids, and nucleotides [1,2]. In 2016, Yoshinori Ohsumi was awarded the Nobel Prize in Physiology or Medicine for his pioneering work in identifying and characterizing the mechanisms of autophagy in yeast [3]. As autophagy research continues to evolve, it has become increasingly evident that this process is crucial in cellular development and differentiation [2]. Further research is essential to discover new therapeutic strategies for a wide range of human diseases and pathophysiological conditions, including infectious [4], autoimmune [4,5], metabolic [6], neurodegenerative [7], cardiovascular [8], rheumatic [9], pulmonary [10] and malignant diseases [11,12], as well as aging [13,14,15]. Therefore, the precise identification of autophagy-related proteins (ATGs) is of great significance for understanding their molecular functions and advancing therapeutic strategies for autophagy-related diseases. Traditionally, ATG identification involves analyzing its physical and chemical properties through wet-lab experiments [16,17,18]. Although these methods remain effective, they are often expensive, time-consuming, or both. Hence, computational methods, especially traditional machine learning methods and the currently popular deep learning methods, have received widespread attention for their capability to identify the ATGs rapidly and efficiently with high throughput.

In recent years, computational models have been designed to identify the ATGs from sequence data [19]. For example, Jiao et al. developed the first predictor of ATGs, termed ATGPred-FL [20], which leveraged the support vector machine (SVM) [21] classifier and an optimal sequence-based feature set derived from the two-step feature selection strategy. Subsequently, Yu et al. proposed an ensemble deep learning framework, EnsembleDL-ATG [22], to improve the identification of ATGs. This framework adopted nine position-specific scoring matrix (PSSM) [23]-based features to extract evolutionary information and represent ATGs. While existing methods performed satisfactorily on the benchmark datasets with high accuracy in identifying the ATGs, they still have several drawbacks. For instance, these models primarily rely on handcrafted features, which refer to manually selected or designed attributes derived from sequences based on biological knowledge, such as amino acid composition (AAC) and physicochemical properties. However, these features often fail to fully capture the intricate patterns and relationships hidden in protein sequences, potentially overlooking crucial information pertinent to autophagic activities. Moreover, the ensemble model, like EnsembleDL-ATG, demands substantial computational resources and may be prone to overfitting due to the complexity of their multiple deep neural network architectures. Therefore, there is still considerable room for enhancing the prediction performance of ATGs.

Recent advancements in natural language processing (NLP) technology have revolutionized various fields, including bioinformatics [24]. Pre-trained protein language models (PLMs) are a class of NLP models that leverage NLP techniques by treating protein sequences as “sentences” [25]. They are trained on extensive protein sequence databases in a self-supervised manner, allowing for the extraction of comprehensive features that rely solely on sequence information. As large-scale protein datasets are increasingly generated, PLMs have become indispensable tools in protein science research [26] and demonstrated remarkable success in a wide range of protein-related downstream tasks, including peptide recognition [27,28], protein subcellular localization [29], protein folding prediction [30], post-translational modification site identification [31,32], and so on [33,34]. However, to the best of our knowledge, these advancements in PLMs and the context-based representation derived from these models have not been explored for predicting ATGs.

**Figure 1:**
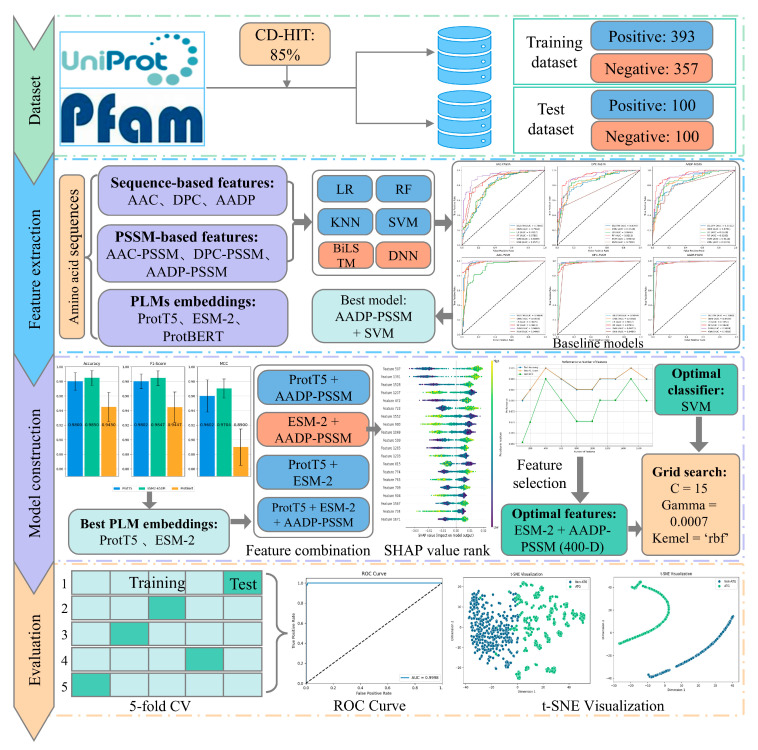
The flow chart of the PLM-ATG model.

In this study, we proposed a novel computational model called PLM-ATG that integrates the SVM classifier with the fusion of PLM embeddings and traditional PSSM-based features to identify ATGs accurately. First, 36 baseline models were established by extracting three types of sequence-based features and three types of PSSM-based features and employing six classifiers, including machine learning algorithms and deep learning architectures. Among these, the SVM classifier with the AADP-PSSM [35] feature set emerged as the top-performing model. Second, we evaluated and compared the performance of three PLM embeddings (i.e., ProtT5 [25], ESM-2 [36], and ProtBERT [25]) by training SVMs on the benchmark dataset. ProtBERT embeddings were excluded due to their lowest accuracies, and the remaining two PLM embeddings were fused with the AADP-PSSM features to further improve the prediction of ATGs. Third, to remove redundant and irrelevant features, we employed Shapley Additive Explanations (SHAP) [37] to select the optimal feature subset from the combination of the ESM-2 embeddings and AADP-PSSM features. The selected features were used to train the final SVM model. Results on the five-fold cross-validation (CV) and the independent test set suggested that the proposed model outperformed existing methods and could serve as a useful tool for the annotation of ATGs, leveraging the fused feature representations. Additionally, we applied t-distributed stochastic neighbor embedding (t-SNE) [38] to visualize the powerful discriminatory ability of PLM-ATG. Figure 1 illustrates the flow chart of the PLM-ATG.

## 2. Results and Discussion

### 2.1. Performance Analysis of Baseline Models

**Figure 2:**
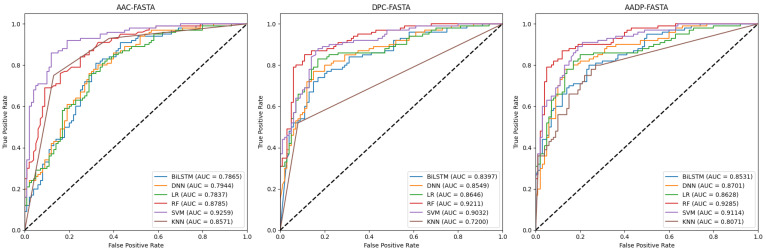
ROC curves for sequence-based models on the independent test set.

**Figure 3:**
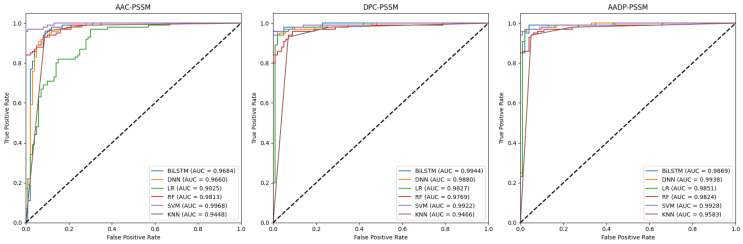
ROC curves for PSSM-based models on the independent test set.

In this section, we employed six classifiers, i.e., logistic regression (LR) [39], random forest (RF) [40], SVM, k-nearest neighbors (KNN) [41], bidirectional long short-term memory (BiLSTM) [42], and deep neural network (DNN) [43], to compare the performance of six traditional feature representations, including AAC, dipeptide composition (DPC), their combination (AADP), AAC-PSSM, DPC-PSSM, and AADP-PSSM for the identification of ATGs. The dataset was randomly divided into two subsets with a ratio of 8:2. The 80% subset is the training set, while the remaining subset is the independent test set to validate the performance of the models. As a result, 36 baseline models were trained on the training set by the five-fold CV, respectively. And their performance was assessed using the following metrics: accuracy (Acc), precision (Pre), sensitivity (Sen), specificity (Spe), F1-score, and Matthews correlation coefficient (MCC) [44,45,46], presented in Table 1 and Table 2. Moreover, the corresponding receiver operating characteristic (ROC) curves and the area under the curve (AUC) values were shown in Figure 2 and Figure 3.

As can be seen from Table 1 and Table 2, PSSM-based features consistently yield superior performance compared to sequence-based features across all models. This enhancement can be attributed to the evolutionary information embedded within PSSM profiles, which could provide some valuable clues for the identification of ATGs. Notably, for sequence-based features, the performance of deep learning models is poorer than that of machine learning models. This indicates that in cases with small datasets, simple features may not fully leverage the potential of deep learning architectures to capture complex relationships and patterns. In addition, the SVM classifier exhibits an outstanding ability to identify the ATGs when combined with each feature, suggesting that the SVM is very efficient and especially suitable for this task. Moreover, the combination of SVM and AADP-PSSM is superior to other models in terms of Acc (0.9750), MCC (0.9500), and Spe (0.9800). Similar conclusions are illustrated in Figure 2 and Figure 3.

### 2.2. Performance Comparison of Three PLM Embeddings

**Figure 4:**
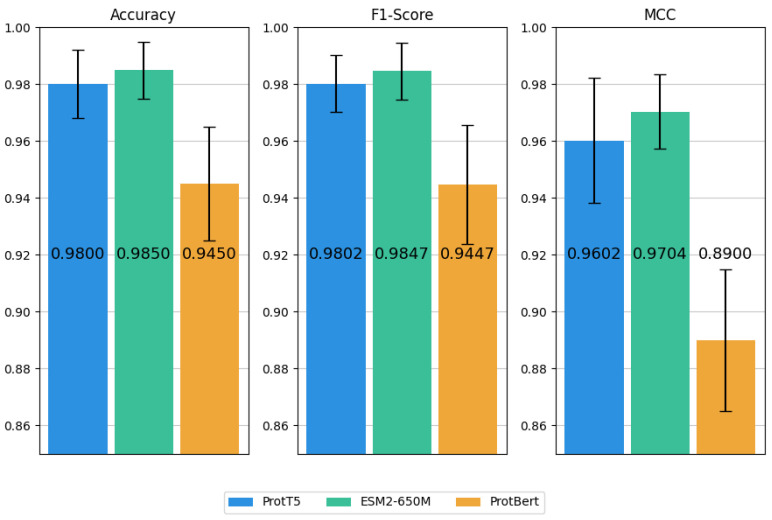
Performance comparison of three PLM embeddings.

To assess and compare the efficacy of the three PLM embeddings in identifying the ATGs, we selected SVM as the sole classifier. This approach allowed us to focus on the relative effectiveness of each PLM embedding, avoiding confounding factors from different classifiers. The SVM classifier performs exceptionally well in handling high-dimensional data, particularly when the number of features exceeds the sample size. Moreover, the SVMs consistently outperform other classifiers in baseline models. The performance of each PLM embedding on the independent test set was visualized in Figure 4.

As depicted in Figure 4, three PLM embeddings exhibit robust performance across all metrics. Specifically, ESM-2 embeddings achieve the best results in terms of Acc (0.9850), F1-score (0.9847), and MCC (0.9704), indicating their superior capacity to distinguish ATGs. Similarly, ProtT5 embeddings show comparable efficacy with marginal performance differences, which suggests they have equivalent potential for this task. In contrast, ProtBERT embeddings underperform the other two PLM embeddings with an Acc of 0.9450, an F1-score of 0.9447, and an MCC of 0.8900. Moreover, we can find that models employing ESM-2 and ProtT5 embeddings surpass all baseline models, while ProtBERT embeddings underperform even conventional PSSM-based features. Based on these findings, ProtT5 and ESM-2 embeddings are identified as the most promising candidates for further analysis in the ATGs identification.

### 2.3. Performance Analysis of Feature Selection

Our experiments have explored the performance of nine distinct feature representations in the ATGs recognition task. The superior performance of the embeddings from ESM-2 and ProtT5, along with the excellent results of AADP-PSSM features, motivates further investigation into the effectiveness of combining these feature representations. The performance of the SVMs employing various feature combinations on the independent test set is summarized in Table 3.

As can be seen from Table 3, the combination of ESM-2 embeddings and AADP-PSSM features achieves better and more stable performance compared to the individual feature representations and other combined feature sets, suggesting the effectiveness of this feature combination strategy. However, incorporating AADP-PSSM features into ProtT5 embeddings only enhances precision, and the overall performance improvement remains marginal. Additionally, the performance of the combined feature sets ProtT5+ESM-2 and ProtT5+ESM-2+AADP-PSSM is inferior to that of the individual PLM embeddings. This indicates that potentially irrelevant or redundant features in these combined representations have an unfavorable impact on the model’s performance.

**Figure 5:**
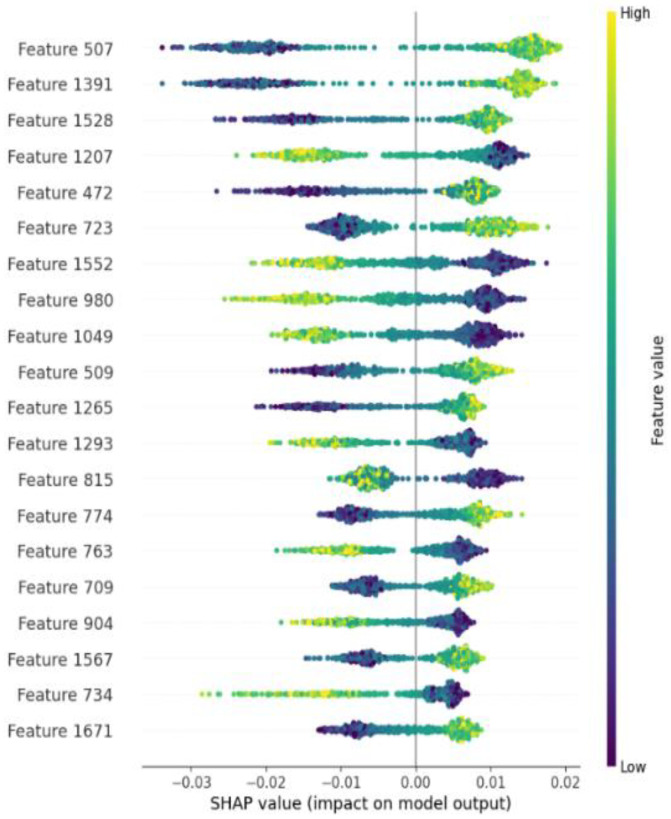
SHAP analysis of the importance of features on ESM-2+AADP-PSSM feature sets.

Although the combined feature set captured more information from multiple aspects, the potential redundant or irrelevant features may lead to the model overfitting and the training time increasing. Therefore, we investigated the impact of feature selection on the performance of the ATG identification. For this experiment, we employed SHAP analysis to quantify the contribution of individual features within the ESM-2 + AADP-PSSM feature set to the model’s output. Figure 5 presents a scatter plot of feature density, where each row represents a feature. Each colored point represents a sample, with yellow indicating higher feature values and purple indicating lower feature values. The horizontal axis shows the SHAP values, and the features are sorted according to the average absolute SHAP value of all samples. Those top-ranking features have a meaningful impact on the model’s output. Conversely, features with low absolute SHAP values are identified as potential sources of redundancy and noise, which may weaken the model’s overall performance.

**Figure 6:**
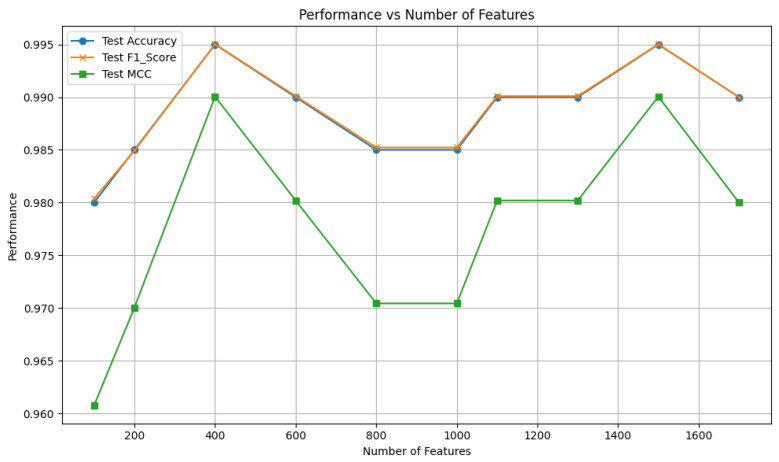
Performance of the SVM with different feature subsets.

After ranking these features according to their SHAP values, we systematically evaluated feature subsets of varying dimensions by inputting the top K features into the SVM classifier to identify the most optimal feature subset, where K = 1700, 1500, 1300, …, 100. The performance of the SVM with different feature subsets on the independent test set was shown in Figure 6, from which we can see that the model achieves peak performance with an accuracy of 99.50% when K = 400 and K = 1500. The 400-D feature subset likely captures highly informative features, which effectively reduces redundancy while retaining crucial patterns. In contrast, the 1500-D feature subset may leverage additional complex patterns because of more features. Considering the trade-off between performance and computational efficiency, the 400-D ESM-S+AADP-PSSM feature subset was finally selected to feed into the SVM classifier for training, resulting in the PLM-ATG model.

**Figure 7:**
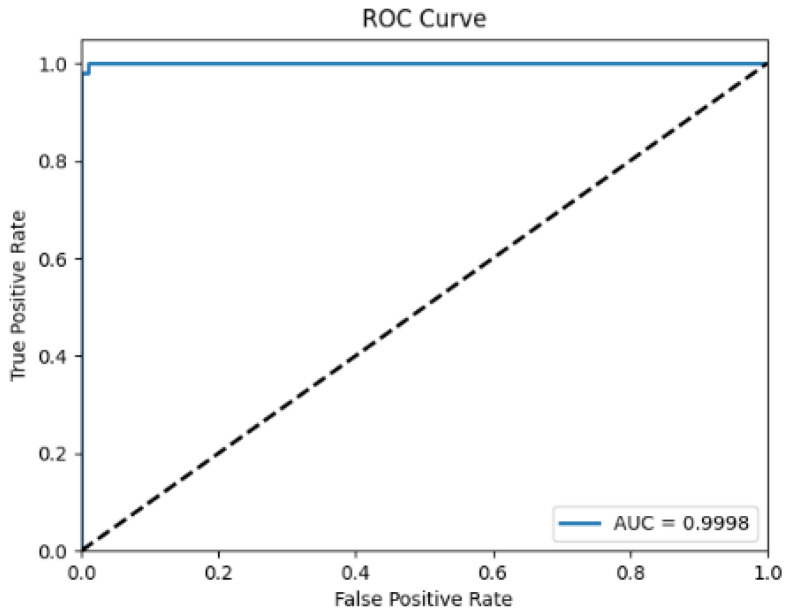
The ROC curves of PLM-ATG on the independent test set.

The performance and generalization ability of the PLM-ATG model were evaluated on the independent test set. The ROC curve presented in Figure 7 shows an excellent AUC value of 0.9998, indicating a near-perfect capability of PLM-ATG to identify ATGs.

### 2.4. Interpretability of the PLM-ATG Model

To further illustrate the effectiveness of the SVM classifier during feature learning and perform a detailed visualization of high-dimensional feature representations learned by the PLM-ATG model, we employed the t-SNE, a widely utilized machine learning algorithm for dimensionality reduction and visualization of high-dimensional data in a lower-dimensional space.

**Figure 8:**
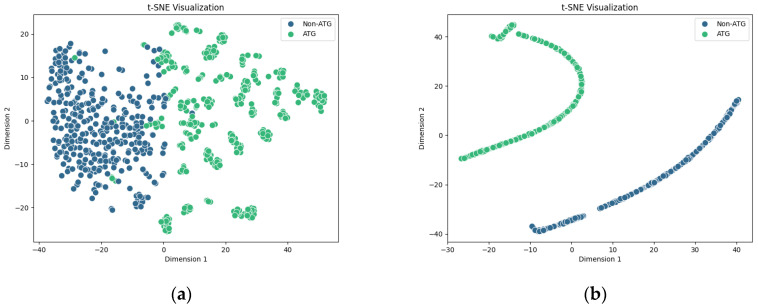
Distribution of ATGs and non-ATGs in the two-dimensional feature space. (**a**) Initial feature space before training; (**b**) Feature space after SVM training.

Figure 8a presents a direct mapping of original features from the 400-D ESM-2+AADP-PSSM feature subset before model training. Although there is a discernible trend of separation between ATGs and non-ATGs, a degree of overlap remains. This suggests that the initial features possess inherent limitations in their discriminative power. In contrast, the distribution of final hidden features extracted by the SVM classifier after training, as shown in Figure 8b, reveals distinctly separated clusters for ATGs and non-ATGs in the two-dimensional space. Visualizing the feature distribution before and after the SVM training demonstrates a reduction in category overlap, indicating that our model successfully learns key information during training.

### 2.5. Performance Comparison with Existing Models

**Figure 9:**
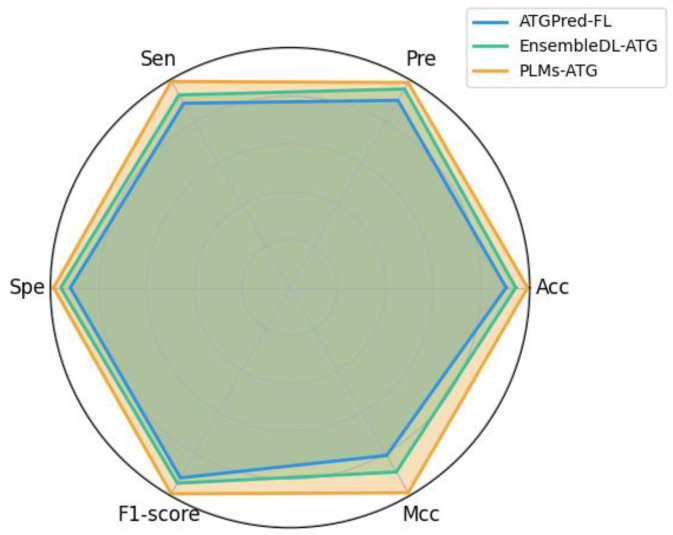
Performance comparison with existing models on the independent test set.

To the best of our knowledge, there are only two computational tools for the identification of ATGs on the same dataset, i.e., ATGPred-FL and EnsembleDL-ATG. As mentioned above, these models relied on a variety of handcrafted features to train machine learning algorithms or deep learning architectures for predicting the ATGs. For a fair comparison with existing methods, we adopted the same training set and independent test set to evaluate the identification performance. The results are visually presented in Figure 9 using the common metrics, including Acc, Pre, Sen, Spe, F1-score, and MCC.

Referring to Figure 9, the proposed PLM-ATG model demonstrates state-of-the-art performance with an Acc of 0.995, Pre of 0.990, Sen of 0.995, Spe of 0.990, F1-score of 0.995, and MCC of 0.990, while achieving significant improvement rates of 5.0–9.0%, 3.1–9.4%, 6.5–10.5%, 3.0–7.0%, 5.1–7.7%, and 10–18% in terms of Acc, Pre, Sen, Spe, F1-score, and MCC relative to existing methods. These comparisons indicate that the PLM embeddings have more exceptional capability for ATG prediction compared with traditional feature representations.

Notably, ATGPred-FL maintains computational efficiency through sequence-based features with SVM classifiers. PLM-ATG similarly employs lightweight SVM, enabling rapid CPU-based predictions. However, ESM-2 embedding extraction requires approximately 8GB RAM during offline preprocessing. In contrast, EnsembleDL-ATG’s CNN-BiLSTM-BiGRU framework demands GPU acceleration throughout both training and predicting, with CPU deployment resulting in substantially higher latency. This indicates that PLM-ATG achieves a balance between performance and computational efficiency, thereby serving as a powerful, efficient, and promising tool for autophagy research.

### 2.6. Web Server Implementation

**Figure 10:**
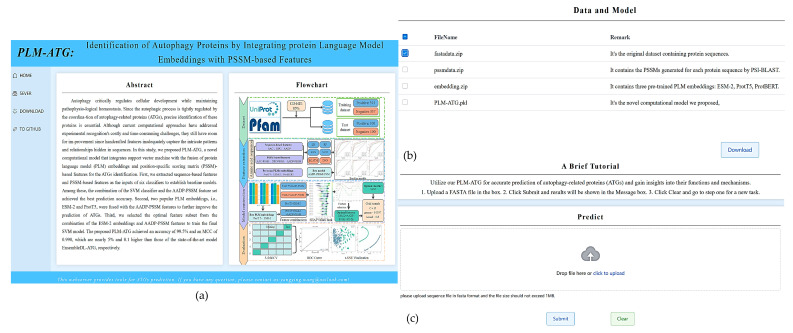
Screenshots of the ATG-PLM web server. (**a**) The web interface; (**b**) data download interface; and (**c**) input data upload and predict interface.

To facilitate the use of our model for ATGs prediction by researchers, we have built a user-friendly web server, which can be publicly accessed at https://www.cciwyy.top (accessed on 30 March 2025), as shown in Figure 10a. Users can upload their query sequence files in FASTA format to the server for prediction. In addition, the datasets, corresponding PSSM data, and PLM embedding data used in this study can be accessed from our online server to validate our findings. If the user needs to obtain more details, he can jump to our open-source repository through “TO GITHUB”.

## 3. Materials and Methods

### 3.1. Datasets

A high-quality benchmark dataset is the critical first step in developing a robust and efficient classification model. In this study, we used the dataset initially constructed by Jiao et al. [20] and subsequently employed by Yu et al. [22]. Specifically, the dataset contains 493 experimentally verified positive samples (ATGs) derived from the latest universal protein knowledgebase (UniProtKB) [47] based on functional annotations and 493 negative samples (non-ATGs) selected from the protein families database (Pfam) [48] based on two principles: removing protein families associated with ATGs and retaining the longest sequences from each remaining protein family. Notably, all homologous sequences were removed using the CD-HIT program [49] with an 85% identity threshold, and non-ATGs were randomly selected from the initial 9788 negative samples to match the positive set size for balance. To ensure consistency and enable meaningful comparisons with previous studies, we adopted the same dataset and randomly segmented 20% of this dataset as an independent test set for unbiased evaluation. The remaining sequences were used for model training and parameter tuning. However, during a thorough data review, we identified 36 duplicate negative samples in the training set. To maintain the integrity of the dataset and improve the accuracy of our model, we removed these duplicate samples in the subsequent study. Detailed information of the benchmark is provided in Table 4.

### 3.2. Feature Representation

#### 3.2.1. PLM Embedding

Leveraging NLP techniques, PLMs are pre-trained on large-scale protein databases, including UniRef50 [50], UniRef100 [51], Big Fantastic Database (BFD) [52,53], and other non-redundant protein datasets. These models not only propelled advancements in the field of proteomics but also opened new avenues in bioinformatics and computational biology, such as ProtT5, ESM-1b [54], ESM-2, ProtBERT, and so on. In this study, we investigated the ability of three PLMs to encode ATGs into vector representations (i.e., embeddings) for ATG identification.

ProtT5, introduced by Google DeepMind, is a protein representation learning framework based on the T5 model [55] whose “text-to-text” architecture enables it to represent different tasks as text generation problems. ProtBERT employs the bidirectional self-attention mechanism of the BERT algorithm [56] to capture long-range dependencies and thoroughly explore contextual information within protein sequences. ESM-2, the second-generation evolutionary scale model introduced by Meta AI [36], leverages multiple sequence alignment (MSA) [57] data to incorporate evolutionary information into its training process. Through self-supervised learning [58], ESM-2 learns deep representations of protein sequences from large-scale evolutionary data, enabling it to generalize effectively to unseen proteins.

We took protein sequences as the input of pre-trained PLMs and directly extracted self-supervised embeddings without fine-tuning. Each protein sequence of length L was transformed into a PLM embedding of size L × N, where N denotes the dimension of the individual embedding for each amino acid. To obtain a uniform feature representation with a fixed dimension, we averaged the individual residue embeddings into a single vector representation for the entire protein. This approach captures the overall protein characteristics rather than focusing on specific residues. Finally, the embeddings generated by ProtT5 and ProtBERT have the same dimension of 1024, while ESM-2 yields the 1280-dimensional embeddings.

#### 3.2.2. Sequence-Based Features

To comprehensively characterize protein sequences from multiple perspectives, a variety of sequence-based features have been designed in previous studies [59,60,61]. In this study, we adopted three widely used features, i.e., AAC, DPC, and AADP.

AAC is defined as a 20-dimensional vector that provides the occurrence numbers of 20 natural amino acids, normalized with a total number of residues in a protein. Clearly, AAC could not reflect the sequence-order information encoded in the protein sequence. To address this issue, DPC was developed to calculate the occurrence probability of contiguous amino acid pairs (dipeptides) within a protein sequence. Since there are 400 possible dipeptide combinations, DPC is represented as a 400-dimensional feature vector. Additionally, AADP is adopted to transform proteins with different lengths into 420-dimensional vectors by concatenating AAC and DPC.

#### 3.2.3. PSSM-Based Features

Previous studies have demonstrated that PSSM-based features can incorporate important evolutionary information, thus enhancing the performance of protein prediction models [62,63,64]. In this study, homologous protein sequences for each protein in the dataset were searched using the PSI-BLAST [23] against the UniRef50 database with three iterations and an E-value cutoff of 0.001. Subsequent multiple sequence alignment generated an initial PSSM of dimensions L×20 for a query protein of length L. In the PSSM, the value of the (i, j)-th element is log-odds score derived from multiple alignment statistics, representing the evolutionary propensity for the amino acid at the i-th position of the query sequence to mutate into residue type j. To reduce bias and noise, the original PSSM are normalized using the following sigmoid function:

(1) $$f\left(s\right)=\frac{1}{1+e^{-s}},$$

where s is the original PSSM value. The resulting PSSM is denoted as:

(2) $$P=\left(\begin{array}{c} \begin{array}{cc} p_{1,1} & p_{1,2}\\ \vdots & \vdots \\ p_{i,1} & p_{i,2} \end{array}\;\;\;\begin{array}{cc} \ldots & p_{1,20}\\ \vdots & \vdots \\ \ldots & p_{i,20} \end{array}\\ \begin{array}{cc} \vdots & \vdots \\ p_{L,1} & p_{L,2} \end{array}\;\;\;\begin{array}{cc} \vdots & \vdots \\ \ldots & p_{L,20} \end{array} \end{array}\right).$$

To convert PSSMs of different proteins into fixed-length feature vectors, the three sequence-based feature extraction methods mentioned above are extended from the primary sequence to the PSSM, yielding corresponding feature representations termed AAC-PSSM, DPC-PSSM, and AADP-PSSM, respectively [35]. Explicitly, they are defined by the following formulas:

(3) $$\mathrm{AAC-PSSM}=\left(x_{1},x_{2},\ldots ,x_{20}\right),$$

(4) $$\mathrm{DPC-PSSM}=\left(y_{1,1},\ldots ,y_{1,20},y_{2,1}\ldots ,y_{2,20},\ldots ,y_{20,1},..,y_{20,20}\right),$$

(5) AADP-PSSM=AAC-PSSM⊕DPC-PSSM,

where

(6) $$x_{j}=\frac{1}{L}\sum_{i=1}^{L}p_{i,j}\left(j=1,2,\ldots ,20\right),$$

(7) $$y_{i,j}=\frac{1}{L-1}\sum_{k=1}^{L-1}p_{k,i}\times p_{k+1,j}\;\left(1\leq i,j\leq 20\right),$$

and ⊕ is the operator of the concatenation.

### 3.3. Model Architecture

In addition to feature extraction, the design of the classification algorithm is also a crucial step that can significantly influence the performance of the model. In this study, we first employed four representative machine learning models to perform the prediction of ATGs, including LR, RF, SVM, and KNN. All models were implemented using the scikit-learn package (v1.5.1) [65]. Their hyperparameters were optimized based on a 5-fold CV using a grid search strategy. Moreover, more specific implementation and parameters can be found on GitHub (https://github.com/YangyingWang/PLM-ATG/tree/main/parameters) (accessed on 30 March 2025).

**Figure 11:**
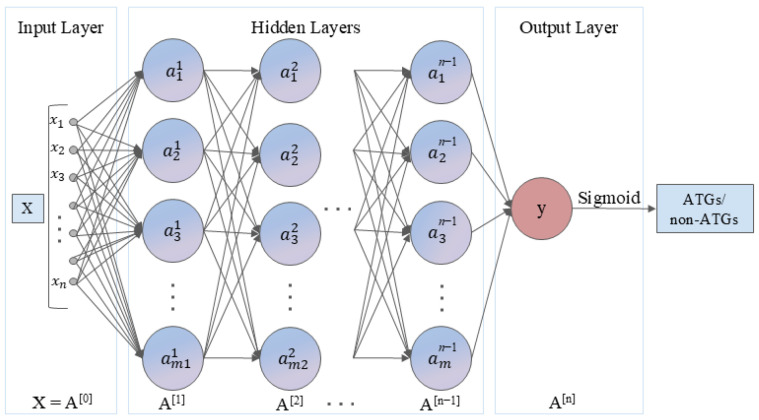
The architecture of DNN.

Moreover, two deep learning algorithms were adopted to conduct the identification of ATGs, including a DNN and a BiLSTM network. DNN is a type of artificial neural network with multiple hidden layers between the input and output layers, capable of learning complex patterns and representations from the input features. Considering significant differences in feature dimensions, we developed two DNN architectures: a lightweight two-layer network (15–10 neurons) for 20-D feature sets, and a deeper three-layer structure (350–300–250 neurons) for 400-D and 420-D feature sets. Moreover, ReLU activation functions were used in the hidden layers to introduce non-linearity, while a dropout rate of 0.2 was applied to prevent overfitting. Binary cross-entropy was used as the loss function. The model was trained using the Adam optimizer with a learning rate of 0.001 and a batch size of 8. Figure 11 shows the DNN architecture.

**Figure 12:**
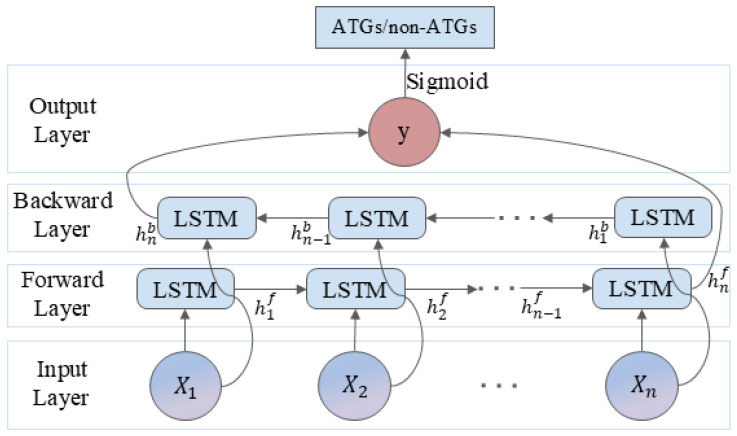
The architecture of BiLSTM.

BiLSTM is a type of recurrent neural network (RNN) [66] architecture that is particularly effective for sequence prediction tasks. It enhances the capability of traditional LSTMs [67] by processing sequences in both forward and backward directions, enabling it to capture context from both ends of a sequence. Our BiLSTM model, comprising two bidirectional LSTM layers, each containing 128 hidden units, was trained using the Adam optimizer with a learning rate of 0.001 and binary cross-entropy loss, with a batch size of 16. Figure 12 shows the BiLSTM architecture.

### 3.4. Performance Evaluation

In this study, the 5-fold CV and an independent test set were performed to evaluate the performance of our models for the identification of ATGs. The predictive ability of the proposed model was assessed by six commonly used metrics: Acc, Pre, Sen, Spe, F1-score, and MCC. These metrics are defined by the following equations:

(8) $$Acc=\frac{TP+TN}{TP+TN+FP+FN},$$

(9) $$Pre=\frac{TP}{TP+FP},$$

(10) $$Sen=\frac{TP}{TP+FN},$$

(11) $$Spe=\frac{TN}{TN+FP},$$

(12) $$F1-\mathrm{score}=\frac{2\times TP}{2\times TP+FP+FN},$$

(13) $$MCC=\frac{TP\times TN-FP\times FN}{\sqrt{\left(TP+FP\right)\times \left(TP+FN\right)\times \left(TN+FP\right)\times \left(TN+FN\right)}}$$

where TP, TN, FP, and FN represent the numbers of true positives, true negatives, false positives, and false negatives, respectively.

Additionally, the area under the receiver operating characteristic (ROC) curve (AUC) was calculated as a useful measure to evaluate the model’s efficacy.

## 4. Conclusions

Advancing computational methods for reliable ATG identification could profoundly impact biomedical research about molecular mechanisms of cellular homeostasis and targeted therapeutic development. In this study, a novel computational model named PLM-ATG was proposed to identify the ATGs. First, we extracted sequence-based and PSSM-based features as the inputs of six classifiers to establish baseline models for reference. Of these models, the combination of the SVM classifier and the AADP-PSSM feature set achieved the best prediction accuracy. Second, two popular PLM embeddings, i.e., ESM-2 and ProtT5, were fused with the AADP-PSSM features to further improve the prediction of ATGs. Third, we selected the optimal feature subset from the combination of the ESM-2 embeddings and AADP-PSSM features to train the final SVM model. Evaluation results on the independent test set demonstrated that the PLM-ATG performs well in the ATG identification task and achieves a significant advancement over existing state-of-the-art models. Thus, the PLM-ATG emerges as a powerful tool for accelerating autophagy research, complemented by an accessible web server implementation at https://www.cciwyy.top (accessed on 30 March 2025), which empowers researchers to leverage the PLM-ATG. Furthermore, the methods and insights presented here provide a valuable reference for the development of predictive models in other protein-related fields, further confirming the transformative impact of NLP technology on bioinformatics. We have made the corresponding data and code publicly available on GitHub (https://github.com/YangyingWang/PLM-ATG) (accessed on 30 March 2025) to facilitate further exploration and development. Although PLM-ATG has achieved perfect performance in identifying ATGs, we believe that future efforts should be directed toward the study of ATGs and their functions.

## Figures and Tables

**Table 1:** Performance of sequence-based models on the independent test set.

Feature	Classifier	Acc	Pre	Sen	Spe	F1-Score	MCC
AAC	LR	0.7150	0.6937	0.7700	0.6600	0.7299	0.4326
	RF	0.7950	0.7706	0.8400	0.7500	0.8038	0.5924
	SVM	0.8450	0.8224	0.8800	0.8100	0.8502	0.6917
	KNN	0.8100	0.8523	0.7500	0.8700	0.7979	0.6245
	BiLSTM	0.7150	0.6720	0.8400	0.5900	0.7467	0.4441
	DNN	0.7250	0.6772	0.8600	0.5900	0.7577	0.4674
DPC	LR	0.8150	0.8247	0.8000	0.8300	0.8122	0.6303
	RF	0.8200	0.7807	0.8900	0.7500	0.8318	0.6464
	SVM	0.8450	0.8224	0.8800	0.8100	0.8502	0.6917
	KNN	0.7200	0.8667	0.5200	0.9200	0.6500	0.4801
	BiLSTM	0.7800	0.8111	0.7300	0.8300	0.7684	0.5628
	DNN	0.7900	0.8295	0.7300	0.8500	0.7766	0.5842
AADP	LR	0.8150	0.8247	0.8000	0.8300	0.8122	0.6303
	RF	0.8400	0.8036	0.9000	0.7800	0.8491	0.6849
	SVM	0.8450	0.8224	0.8800	0.8100	0.8502	0.6917
	KNN	0.6900	0.8276	0.4800	0.9000	0.6076	0.4187
	BiLSTM	0.7600	0.8250	0.6600	0.8600	0.7333	0.5307
	DNN	0.8000	0.8659	0.7100	0.8900	0.7802	0.6100

**Table 2:** Performance of PSSM-based models on the independent test set.

Feature	Classifier	Acc	Pre	Sen	Spe	F1-Score	MCC
AAC-PSSM	LR	0.8250	0.7826	0.9000	0.7500	0.8372	0.6574
	RF	0.9200	0.9200	0.9200	0.9200	0.9200	0.8400
	SVM	0.9700	0.9700	0.9700	0.9700	0.9700	0.9400
	KNN	0.9350	0.9143	0.9600	0.9100	0.9366	0.8711
	BiLSTM	0.9300	0.9057	0.9600	0.9000	0.9320	0.8616
	DNN	0.9200	0.8889	0.9600	0.8800	0.9231	0.8427
DPC-PSSM	LR	0.9350	0.9065	0.9700	0.9000	0.9372	0.8721
	RF	0.9350	0.9307	0.9400	0.9300	0.9353	0.8700
	SVM	0.9700	0.9796	0.9600	0.9800	0.9697	0.9402
	KNN	0.9300	0.9300	0.9300	0.9300	0.9300	0.8600
	BiLSTM	0.9650	0.9515	0.9800	0.9500	0.9500	0.9304
	DNN	0.9400	0.9783	0.9000	0.9800	0.9375	0.8828
AADP-PSSM	LR	0.9500	0.9327	0.9700	0.9300	0.9510	0.9007
	RF	0.9400	0.9314	0.9500	0.9300	0.9406	0.8802
	SVM	0.9750	0.9798	0.9700	0.9800	0.9749	0.9500
	KNN	0.9450	0.9495	0.9400	0.9500	0.9447	0.8900
	BiLSTM	0.9750	0.9612	0.9900	0.9600	0.9754	0.9504
	DNN	0.9450	0.9238	0.9700	0.9200	0.9463	0.8911

**Table 3:** Performance comparison of feature combinations on the independent test set.

Feature	Dimension	Acc	Pre	Sen	Spe	F1-Score	MCC
ProtT5	1024	0.9800	0.9706	0.9900	0.9700	0.9802	0.9602
ESM-2	1280	0.9850	0.9900	0.9700	0.9900	0.9848	0.9704
ProtT5 + AADP-PSSM	1444	0.9800	0.9898	0.9700	0.9900	0.9798	0.9602
ESM-2 + AADP-PSSM	1700	0.9900	0.9900	0.9900	0.9900	0.9900	0.9800
ProtT5 + ESM-2	2304	0.9800	0.9706	0.9900	0.9700	0.9802	0.9602
ProtT5 + ESM-2 + AADP-PSSM	2724	0.9800	0.9706	0.9900	0.9700	0.9802	0.9602

**Table 4:** Description of the training and independent test sets.

Dataset Type	Positive (ATGs)	Negative (Non-ATGs)
Training	393	357
Independent test	100	100

## Data Availability

The data and the source code used to support the findings of this study are freely available to the academic community at https://github.com/YangyingWang/PLM-ATG, accessed on 5 March 2025.

## References

[1] Cuervo A.M. (2004). Autophagy: In sickness and in health. Trends Cell Biol..

[2] Levine B., Klionsky D.J. (2004). Development by self-digestion: Molecular mechanisms and biological functions of autophagy. Dev. Cell.

[3] Levine B., Klionsky D.J. (2017). Autophagy wins the 2016 Nobel Prize in Physiology or Medicine: Breakthroughs in baker’s yeast fuel advances in biomedical research. Proc. Natl. Acad. Sci. USA.

[4] Deretic V., Saitoh T., Akira S. (2013). Autophagy in infection, inflammation and immunity. Nat. Rev. Immunol..

[5] Zhong Z., Sanchez-Lopez E., Karin M. (2016). Autophagy, Inflammation, and Immunity: A Troika Governing Cancer and Its Treatment. Cell.

[6] Kim K.H., Lee M.-S. (2014). Autophagy-a key player in cellular and body metabolism. Nat. Rev. Endocrinol..

[7] Menzies F.M., Fleming A., Rubinsztein D.C. (2015). Compromised autophagy and neurodegenerative diseases. Nat. Rev. Neurosci..

[8] Shirakabe A., Ikeda Y., Sciarretta S., Zablocki D.K., Sadoshima J. (2016). Aging and Autophagy in the Heart. Circ. Res..

[9] Rockel J.S., Kapoor M. (2016). Autophagy: Controlling cell fate in rheumatic diseases. Nat. Rev. Rheumatol..

[10] Nakahira K., Porras M.A.P., Choi A.M.K. (2016). Autophagy in Pulmonary Diseases. Am. J. Respir. Crit. Care Med..

[11] Amaravadi R., Kimmelman A.C., White E. (2016). Recent insights into the function of autophagy in cancer. Genes Dev..

[12] Galluzzi L., Bravo-San Pedro J.M., Demaria S., Formenti S.C., Kroemer G. (2017). Activating autophagy to potentiate immunogenic chemotherapy and radiation therapy. Nat. Rev. Clin. Oncol..

[13] Meléndez A., Tallóczy Z., Seaman M., Eskelinen E.L., Hall D.H., Levine B. (2003). Autophagy genes are essential for dauer development and life-span extension in *C. elegans*. Science.

[14] Lapierre L.R., Kumsta C., Sandri M., Ballabio A., Hansen M. (2015). Transcriptional and epigenetic regulation of autophagy in aging. Autophagy.

[15] Lopez-Otin C., Galluzzi L., Freije J.M.P., Madeo F., Kroemer G. (2016). Metabolic Control of Longevity. Cell.

[16] Jiang P., Mizushima N. (2015). LC3-and p62-based biochemical methods for the analysis of autophagy progression in mammalian cells. Methods.

[17] Mizushima N., Yoshimori T., Levine B. (2010). Methods in mammalian autophagy research. Cell.

[18] Martinet W., Timmermans J.-P., De Meyer G.R. (2014). Methods to assess autophagy in situ—Transmission electron microscopy versus immunohistochemistry. Methods in Enzymology.

[19] Cheng L., Zeng Y., Hu S., Zhang N., Cheung K.C.P., Li B., Leung K.-S., Jiang L. (2021). Systematic prediction of autophagy-related proteins using *Arabidopsis thaliana* interactome data. Plant J..

[20] Jiao S., Chen Z., Zhang L., Zhou X., Shi L. (2022). ATGPred-FL: Sequence-based prediction of autophagy proteins with feature representation learning. Amino Acids.

[21] Ben-Hur A., Ong C.S., Sonnenburg S., Schoelkopf B., Raetsch G. (2008). Support Vector Machines and Kernels for Computational Biology. PLoS Comput. Biol..

[22] Yu L., Zhang Y., Xue L., Liu F., Jing R., Luo J. (2023). EnsembleDL-ATG: Identifying autophagy proteins by integrating their sequence and evolutionary information using an ensemble deep learning framework. Comput. Struct. Biotechnol. J..

[23] Altschul S.F., Madden T.L., Schaffer A.A., Zhang J.H., Zhang Z., Miller W., Lipman D.J. (1997). Gapped BLAST and PSI-BLAST: A new generation of protein database search programs. Nucleic Acids Res..

[24] Zeng Z., Shi H., Wu Y., Hong Z. (2015). Survey of Natural Language Processing Techniques in Bioinformatics. Comput. Math. Methods Med..

[25] Elnaggar A., Heinzinger M., Dallago C., Rehawi G., Wang Y., Jones L., Gibbs T., Feher T., Angerer C., Steinegger M. (2022). ProtTrans: Toward Understanding the Language of Life Through Self-Supervised Learning. IEEE Trans. Pattern Anal. Mach. Intell..

[26] Asgari E., Mofrad M.R.K. (2015). Continuous Distributed Representation of Biological Sequences for Deep Proteomics and Genomics. PLoS ONE.

[27] Du Z., Ding X., Hsu W., Munir A., Xu Y., Li Y. (2024). pLM4ACE: A protein language model based predictor for antihypertensive peptide screening. Food Chem..

[28] Han J., Kong T., Liu J. (2024). PepNet: An interpretable neural network for anti-inflammatory and antimicrobial peptides prediction using a pre-trained protein language model. Commun. Biol..

[29] Thumuluri V., Armenteros J.J.A., Johansen A.R., Nielsen H., Winther O. (2022). DeepLoc 2.0: Multi-label subcellular localization prediction using protein language models. Nucleic Acids Res..

[30] Villegas-Morcillo A., Gomez A.M., Sanchez V. (2022). An analysis of protein language model embeddings for fold prediction. Brief. Bioinform..

[31] Qi D., Song C., Liu T. (2024). PreDBP-PLMs: Prediction of DNA-binding proteins based on pre-trained protein language models and convolutional neural networks. Anal. Biochem..

[32] Zhang L., Liu T. (2024). PDNAPred: Interpretable prediction of protein-DNA binding sites based on pre-trained protein language models. Int. J. Biol. Macromol..

[33] Li Z., Jin J., Long W., Wei L. (2023). PLPMpro: Enhancing promoter sequence prediction with prompt-learning based pre-trained language model. Comput. Biol. Med..

[34] Medina-Ortiz D., Contreras S., Fernandez D., Soto-Garcia N., Moya I., Cabas-Mora G., Olivera-Nappa A. (2024). Protein Language Models and Machine Learning Facilitate the Identification of Antimicrobial Peptides. Int. J. Mol. Sci..

[35] Liu T., Zheng X., Wang J. (2010). Prediction of protein structural class for low-similarity sequences using support vector machine and PSI-BLAST profile. Biochimie.

[36] Lin Z., Akin H., Rao R., Hie B., Zhu Z., Lu W., dos Santos Costa A., Fazel-Zarandi M., Sercu T., Candido S. (2022). Language models of protein sequences at the scale of evolution enable accurate structure prediction. BioRxiv.

[37] Lundberg S.M., Lee S.-I. A Unified Approach to Interpreting Model Predictions. Proceedings of the 31st Annual Conference on Neural Information Processing Systems (NIPS).

[38] van der Maaten L., Hinton G. (2008). Visualizing Data using t-SNE. J. Mach. Learn. Res..

[39] Boateng E.Y., Abaye D.A. (2019). A review of the logistic regression model with emphasis on medical research. J. Data Anal. Inf. Process..

[40] Cutler D.R., Edwards T.C., Beard K.H., Cutler A., Hess K.T., Gibson J., Lawler J.J. (2007). Random forests for classification in ecology. Ecology.

[41] Cover T., Hart P. (1967). Nearest neighbor pattern classification. IEEE Trans. Inf. Theory.

[42] Zhou P., Shi W., Tian J., Qi Z., Li B., Hao H., Xu B. Attention-based bidirectional long short-term memory networks for relation classification. Proceedings of the 54th Annual Meeting of the Association for Computational Linguistics, (*Volume 2: Short Papers*).

[43] Dhanuka R., Singh J.P., Tripathi A. (2023). A Comprehensive Survey of Deep Learning Techniques in Protein Function Prediction. IEEE-Acm Trans. Comput. Biol. Bioinform..

[44] Lv H., Dao F.-Y., Zulfiqar H., Su W., Ding H., Liu L., Lin H. (2021). A sequence-based deep learning approach to predict CTCF-mediated chromatin loop. Brief. Bioinform..

[45] Jiang Q., Wang G., Jin S., Li Y., Wang Y. (2013). Predicting human microRNA-disease associations based on support vector machine. Int. J. Data Min. Bioinform..

[46] Huang Y., Zhou D., Wang Y., Zhang X., Su M., Wang C., Sun Z., Jiang Q., Sun B., Zhang Y. (2020). Prediction of transcription factors binding events based on epigenetic modifications in different human cells. Epigenomics.

[47] (2021). UniProt: The universal protein knowledgebase in 2021. Nucleic Acids Res..

[48] Bateman A., Coin L., Durbin R., Finn R.D., Hollich V., Griffiths-Jones S., Khanna A., Marshall M., Moxon S., Sonnhammer E.L. (2004). The Pfam protein families database. Nucleic Acids Res..

[49] Fu L., Niu B., Zhu Z., Wu S., Li W. (2012). CD-HIT: Accelerated for clustering the next-generation sequencing data. Bioinformatics.

[50] Suzek B.E., Wang Y., Huang H., McGarvey P.B., Wu C.H., UniProt C. (2015). UniRef clusters: A comprehensive and scalable alternative for improving sequence similarity searches. Bioinformatics.

[51] Suzek B.E., Huang H., McGarvey P., Mazumder R., Wu C.H. (2007). UniRef: Comprehensive and non-redundant UniProt reference clusters. Bioinformatics.

[52] Collobert R., Weston J., Bottou L., Karlen M., Kavukcuoglu K., Kuksa P. (2011). Natural Language Processing (Almost) from Scratch. J. Mach. Learn. Res..

[53] Tran C., Khadkikar S., Porollo A. (2023). Survey of Protein Sequence Embedding Models. Int. J. Mol. Sci..

[54] Meier J., Rao R., Verkuil R., Liu J., Sercu T., Rives A. Language models enable zero-shot prediction of the effects of mutations on protein function. Proceedings of the 35th Annual Conference on Neural Information Processing Systems (NeurIPS).

[55] Raffel C., Shazeer N., Roberts A., Lee K., Narang S., Matena M., Zhou Y., Li W., Liu P.J. (2020). Exploring the Limits of Transfer Learning with a Unified Text-to-Text Transformer. J. Mach. Learn. Res..

[56] Devlin J., Chang M.-W., Lee K., Toutanova K., Assoc Computat L. BERT: Pre-training of Deep Bidirectional Transformers for Language Understanding. Proceedings of the Conference of the North-American-Chapter of the Association-for-Computational-Linguistics—Human Language Technologies (NAACL-HLT).

[57] Rao R., Liu J., Verkuil R., Meier J., Canny J.F., Abbeel P., Sercu T., Rives A. MSA Transformer. Proceedings of the International Conference on Machine Learning (ICML).

[58] Rao R., Meier J., Sercu T., Ovchinnikov S., Rives A. (2020). Transformer protein language models are unsupervised structure learners. Biorxiv.

[59] Zhang Y., Yu S., Xie R., Li J., Leier A., Marquez-Lago T.T., Akutsu T., Smith A.I., Ge Z., Wang J. (2020). PeNGaRoo, a combined gradient boosting and ensemble learning framework for predicting non-classical secreted proteins. Bioinformatics.

[60] Zhang D., Xu Z.-C., Su W., Yang Y.-H., Lv H., Yang H., Lin H. (2021). iCarPS: A computational tool for identifying protein carbonylation sites by novel encoded features. Bioinformatics.

[61] Liu J., Su R., Zhang J., Wei L. (2021). Classification and gene selection of triple-negative breast cancer subtype embedding gene connectivity matrix in deep neural network. Brief. Bioinform..

[62] Luo J., Yu L., Guo Y., Li M. (2012). Functional classification of secreted proteins by position specific scoring matrix and auto covariance. Chemom. Intell. Lab. Syst..

[63] Yu L., Liu F., Li Y., Luo J., Jing R. (2021). DeepT3_4: A Hybrid Deep Neural Network Model for the Distinction Between Bacterial Type III and IV Secreted Effectors. Front. Microbiol..

[64] Yu L., Xue L., Liu F., Li Y., Jing R., Luo J. (2022). The applications of deep learning algorithms on in silico druggable proteins identification. J. Adv. Res..

[65] Pedregosa F., Varoquaux G., Gramfort A., Michel V., Thirion B., Grisel O., Blondel M., Prettenhofer P., Weiss R., Dubourg V. (2011). Scikit-learn: Machine Learning in Python. J. Mach. Learn. Res..

[66] Liu X. (2017). Deep Recurrent Neural Network for Protein Function Prediction from Sequence. arXiv.

[67] Yu Y., Si X., Hu C., Zhang J. (2019). A review of recurrent neural networks: LSTM cells and network architectures. Neural Comput..

